# PARP Inhibitors in First-Line Therapy of Ovarian Cancer: Are There Any Doubts?

**DOI:** 10.3389/fonc.2020.00782

**Published:** 2020-06-12

**Authors:** Elisena Franzese, Anna Diana, Sara Centonze, Sandro Pignata, Ferdinando De Vita, Fortunato Ciardiello, Michele Orditura

**Affiliations:** ^1^Division of Medical Oncology, Department of Precision Medicine, School of Medicine, “Luigi Vanvitelli” University of Campania, Naples, Italy; ^2^Istituto Nazionale per lo Studio e la Cura dei Tumori “Fondazione G. Pascale,” IRCCS, Naples, Italy

**Keywords:** ovarian cancer, first line, BRCA 1/2 mutation carriers, homologous recombination, PARP inhibitor (PARPi)

## Abstract

The standard of care for newly diagnosed advanced ovarian cancer (NADOC) is represented by surgical debulking followed by systemic platinum–taxanes combination chemotherapy. At the last European Society for Medical Oncology (ESMO) Congress, results from three trials testing three different poly-adenosine-diphosphate-ribose-polymerase (PARP) inhibitors (olaparib, niraparib, veliparib) in first-line therapy of OC have been presented. For the first time, these studies evaluated the efficacy of PARP inhibitors in this setting and the relative predictive biomarkers for patients' selection. The use of a PARP inhibitor is related with prolonged progression free survival (PFS) in the whole population of NADOC, although the magnitude of benefit varies widely among subgroups, highlighting the need to identify specific biological subtypes into clinical practice. In this minireview, we discuss the updated data available from clinical studies in this scenario.

## Introduction

The standard of care for newly diagnosed advanced ovarian cancer (NADOC) is represented by surgical debulking followed by systemic platinum–taxanes combination chemotherapy.

The addition of bevacizumab to first-line chemotherapy improves progression free survival (PFS) in patients with higher risk of recurrence (International Federation of Gynecology and Obstetrics FIGO stage IV or suboptimally debulked stage III ovarian cancer—OC). Maintenance therapy with a poly-adenosine-diphosphate-ribose-polymerase (PARP) inhibitor, olaparib, after response to first-line chemotherapy, prolongs PFS in *BRCA*-mutated patients (1).

At the last European Society for Medical Oncology (ESMO) Congress, results from three trials testing three different PARP inhibitors in this clinical setting of OC have been presented. First, the phase III PAOLA-1/ENGOT-OV25 study, combination of PARP-inhibitor olaparib and anti-VEGF bevacizumab, was tested, for the first time, as maintenance therapy after platinum-based chemotherapy obtaining a significant improvement of PFS compared to placebo plus bevacizumab in the overall population regardless of the *BRCA* status (2). Second, the phase III PRIMA/ENGOT-OV26/GOG-3012 trial showed that a different PARP inhibitor, niraparib, used as maintenance therapy also increases PFS vs. placebo in the overall population of OC patients, irrespectively of *BRCA* and homologous recombination deficiency (HRD) status (3). Finally, the phase III VELIA GOG-3005 trial evaluated the addition of the PARP inhibitor veliparib to standard chemotherapy in first line followed by veliparib as maintenance therapy: the triple combination resulted in a significantly longer PFS across the entire population of NADOC, regardless of any biomarker, type of surgery, or associated paclitaxel regimen (4).

For the first time, these studies evaluated the efficacy of PARP inhibitors in first-line therapy of OC and the relative predictive biomarkers for patients' selection. We reviewed the updated data available from clinical studies in this setting highlighting the intrinsic differences among trials (see Table 1).

**Table 1:** Intrinsic characteristics of trials of first-line PARP inhibitors.

**Trial (Number of enrolled patients)**	**Disease subtype (stage at diagnosis)**	**Percentage of biomarker subgroups**	**Premaintenance phase treatment characteristics**	**Randomization and treatment (investigational and comparator arms)**	**Treatment duration**	**Median follow-up duration (investigational and comparator arms)**	**Primary end point**
SOLO1 (391 Pts)	Serous or endometrioid carcinomas *BRCA mut* Stage III: 83% Stage IV: 17%	gBRCA: 388/391 sBRCA: 2/391 BRCA1: 72% BRCA2: 27% Both BRCA1/2: 1.2%	PDS: 65% NACT: 35% CR: 81% PR: 18%	2:1 Olaparib 300 mg BID maintenance PLB	Until disease progression or up to 2 years	40.7 months 41.2 months	Investigator assessed PFS
PRIMA (733 Pts)	Serous or endometrioid carcinomas Stage III: 65% Stage IV: 35%	HRD: 51% BRCAmut: 30% BRCA wt: 20% HRDp:34% HRDnd:15%	PDS: 33% NACT: 67% CR: 69% PR: 31%	2:1 Niraparib 200 mg or 300 mg once daily maintenance PLB maintenance	Until disease progression or up to 3 years	13.8 months	BICR-assessed PFS
PAOLA-1 (806 Pts)	Serous or endometrioid carcinomas Stage III: 70% Stage IV: 29%	HRD positive: 48% HRD positive ex. tBRCAmut:19% tBRCAmut:29% HRDnegative:34%	PDS: 50% NACT: 42% NED: 53% CR: 20% PR: 27%	2:1 Olaparib 300 mg BID + bevacizumab maintenance PLB + bevacizumab maintenance	Until disease progression or up to 15 months (bevacizumab) or 2-years (olaparib)	24.0 months 22.7 months	Investigator-assessed PFS
VELIA (1140 Pts)	Serous carcinomas Stage III: 78% Stage IV: 22%	HRD unknow:18% gBRCA 19% sBRCA 8% HRD: 56% Non-HRD: 33%	PDS 67% NACT: 29%	1:1:1 CBDCA+ PAX +veliparib (150 mg BID) PLB or veliparib maintenance (400 mg BIDe) CBDCA+ PAX + PLB PLB maintenance	Until disease progression or up to 3 years	28 months	Investigator-assessed PFS

*HRDnd, homologous recombination deficiency not determinated; PDS, primary debulking surgery; NACT, neoadjuvant chemotherapy; CR, complete response; PR, partial response; NED, no evidence disease; PLB, placebo; BID, twice daily; PFS, progression-free survival; FIGO, International Federation of Gynecology and Obstetrics; BICR, blinded independent central review; HRd, homologous recombination deficient; HRDp, homologous recombination proficient; HRD, homologous recombination deficiency; tBRCA, tissue-based BRCA; BRCAmut, mutated BRCA; gBRCA, germline BRCA; sBRCA, somatic BRCA; ex, excluding; pts, patients*.

## SOLO-1 and PRIMA Trials: Single Agent PARP Inhibitor as Maintenance Therapy

The SOLO-1 trial was the first trial evaluating the efficacy of maintenance therapy with the PARP inhibitor olaparib in patients with NADOC with a germline or somatic mutation in *BRCA1/2*, who responded to platinum-based chemotherapy (1). The PRIMA/ENGOTOV26/GOG-3012 trial investigated the efficacy and safety of the PARP inhibitor niraparib as maintenance therapy after a response to platinum-based chemotherapy in high-risk NADOC patients (3).

Noteworthy, some clinical differences exist between the SOLO-1 and PRIMA populations.

In fact, SOLO-1 trial enrolled 391 patients subjected to debulking surgery including 85% of stage III FIGO and 15% of stage IV FIGO, with the majority of the patients with no residual macroscopic disease after cytoreductive surgery (76 and 81% in the two groups, respectively) and complete radiological response to chemotherapy reported in 85% of the patients.

In the PRIMA trial, 733 patients at high risk for progressive disease were enrolled: 65% of stage III with visible residual disease after primary debulking surgery and 35% in stage IV FIGO. Thus, stage III FIGO OC patients with no visible residual disease were excluded. However, any residual tumor after completion of chemotherapy had to measure ≤ 2 cm and either CA-125 in the normal range or CA-125 that decreased by more than 90% during their front-line therapy was required. Moreover, among the 67% of patients receiving neoadjuvant chemotherapy, only 31% obtained partial response.

Secondly, genomic analysis and relative patients' stratification were performed with different methods in the two studies. In the SOLO-1 trial, Myriad test was used to confirm the centrally germline *BRCA1/2* mutation status, while the tumor *BRCA1/2* mutation status was assessed retrospectively using a Foundation Medicine platform. These analyses revealed that 388/391 of enrolled patients carried a germline mutation in the *BRCA*1/2 gene, 1 had a *BRCA* variant of uncertain significance, and furthermore 2 had somatic *BRCA* mutations. Conversely, in the PRIMA trial, the tissue homologous recombination (HR) test by Myriad My Choice was used to stratify the population in deficient, proficient, or not determined HR status groups. Out of the 733 enrolled patients, 373 were HRD, 30% had *BRCA* mutations, and 20% were *BRCA* wild type. A total of 249 patients (34%) had HR proficient (HRp) tumors, while for 111 patients (15%), the HRD status was not determined. Thus, SOLO-1 patients were more likely to respond to a PARP inhibitor since the study population was *BRCA* mutated.

Finally, the schedule of treatment was different: in the SOLO-1 trial, olaparib maintenance at a dose of 300 mg twice daily was started after the completion of platinum-based chemotherapy and continued until radiological disease progression, until unacceptable toxicity, or for up to 2 years if no radiological evidence of the disease was achieved. Instead, niraparib maintenance at a dose of 300 mg once daily in the PRIMA trial was scheduled to begin within 12 weeks from the end of chemotherapy and was protracted for 3 years or until disease progression. For patients with ≤ 155 platelets × 10^9^/L and weighing ≤ 77 kg, the emended dose of niraparib was 200 mg once daily.

The primary endpoint of both trials was PFS, and in the PRIMA trial, this endpoint was measured according to a hierarchical test, first in patients with HRD tumors and subsequently in the overall population. Furthermore, the PRIMA trial used exclusively an independent radiologic review to define the disease progression.

The median PFS was not reached in the olaparib arm vs. 13.8 months, while the PFS at 3 years was 60 and 27% for experimental and placebo arm, respectively. Therefore, the SOLO-1 trial demonstrated that olaparib maintenance therapy reduces the risk of disease progression or death of 70% (HR = 0.30; 95% CI 0.23–0.41) in patients with *BRCA*-mutated NADOC. Moreover, updated data are already available from the SOLO-1 trial: the rate of freedom from the use of a second subsequent therapy and from death at 3 years was 74% in the olaparib group and 56% in the placebo group. As subsequent second-line therapy, 33/94 (35%) patients in the placebo arm and 10/91 (11%) patients in the olaparib arm received a PARP inhibitor. The most common adverse events (AEs) with olaparib treatment were fatigue (63.5%), anemia (39%), and nausea (77%). Anemia was frequently reported as a serious AE in about 7% of patients treated with olaparib representing the main cause of treatment discontinuation.

In the PRIMA trial, the median duration of PFS in patients with HRD was 21.9 and 10.4 months in the niraparib and placebo groups (HR=0.43; 95% CI 0.31–0.59), respectively. No significant difference in the benefit obtained by niraparib therapy was observed between patients with HR deficient *BRCA* mutated (22.1 in the niraparib group vs. 10.9 months in the placebo group; HR = 0.40; 95% CI 0.27–0.62) and patients with HR deficient *BRCA* wild type (19.6 in the niraparib group vs. 8.2 months in the placebo group; HR = 0.50; 95% CI 0.31–0.83). Instead, niraparib induces a clinically significant benefit with a 32% risk reduction in progression in HRp patients (8.1 in the niraparib group vs. 5.4 months in the placebo group; HR = 0.68; 95% CI, 0.49–0.94) at 24 months with 81% of survival probability vs. 59% observed in the placebo group (HR = 0.51; 95% CI, 0.27–0.97). These considerations can be mainly explained by differences of population enrolled in two studies, as discussed before. Hematologic AEs resulted higher during niraparib treatment vs. placebo arm: anemia (31.0 vs. 1.6%), thrombocytopenia (28.7 vs. 0.4%), and neutropenia (12.8 vs. 1.2%). Notably, thrombocytopenia led to discontinuation in 4.3% of the cases.

## VELIA and PAOLA-1 trials: Combinations Including PARP Inhibitor in First-Line Therapy

VELIA/GOG-3005 is the first phase III study that evaluated the synergistic combination of the PARP inhibitor veliparib with platinum-based chemotherapy in OC (4).

A total of 1,140 stage III/IV FIGO NADOC patients were randomized to receive (a) chemotherapy plus placebo followed by placebo maintenance (control arm), (b) chemotherapy plus veliparib followed by placebo maintenance (veliparib combination only arm), or (c) chemotherapy plus veliparib followed by veliparib maintenance (veliparib throughout arm). Combination chemotherapy consisted of 6 cycles and maintenance therapy consisted of 30 additional cycles. Veliparib was administrated at a dose of 150 mg orally during chemotherapy, and it was doubled when administered as a single agent. If this dose was not associated with limiting side effects, it was increased to 400 mg daily. Interestingly, veliparib was used at a dose intensity of 37.5% when administered with chemotherapy and carboplatin was used at the standard doses of AUC 6, with high proportion of patients (84 to 93%) completing all planned chemotherapy doses.

Noteworthy, in the VELIA trial, the PFS was measured from the start of frontline chemotherapy, and also patients with stable disease (not only responders) received single-agent veliparib maintenance.

PFS in the “veliparib throughout arm” compared with the “control arm” was the primary endpoint, and it was analyzed sequentially in the *BRCA*-mutation cohort, in the HRD cohort, and the intention-to-treat (ITT) population. In the *BRCA-*mutated cohort, PFS was 34.7 vs. 22.0 months (HR = 0.44; 95% CI 0.28–0.68); in the HRD cohort, PFS was 31.9 vs. 20.5 months (HR = 0.57; 95% CI 0.43–0.76); in the ITT population, PFS was 23.5 vs. 17.3 months (HR = 0.68; 95% CI 0.56–0.83), respectively.

The secondary endpoint was PFS in the “veliparib combination only arm” vs. “control arm.” No benefit in terms of PFS was obtained in any molecular cohort with this schedule.

These results suggest that the benefit from veliparib derives from the maintenance therapy, as seen in the “veliparib-throughout group.” However, the trial did not include an arm with veliparib single-agent maintenance after chemotherapy because it was designed before the clinical data of PARP inhibitors in the context of maintenance therapy had been established. This feature does not allow to clearly evaluate on the contribution of concurrent plus maintenance veliparib therapy in the veliparib-throughout group. Furthermore, an exploratory analysis showed a very slight PFS benefit in the non-mutated *BRCA* subgroup (i.e., HRD tumors with non-mutated *BRCA*; HR 0.80; 95% CI, 0.64–0.99) compared to the non-HRD cohort of patients (true non-mutated *BRCA* status; HR 95% CI, 0.60–1.09) of the veliparib-throughout arm.

Few other trials explored combinations of PARP inhibitors and chemotherapy in the treatment of OC (5, 6). Among them, the randomized phase 2 trial comparing olaparib combined with paclitaxel and carboplatin vs. chemotherapy alone in recurrent platinum-sensitive OC resulted in significantly improved PFS of combination arm, especially in *BRCA*-mutated patients (7). However, to guarantee a more manageable tolerability profile, in this study, carboplatin dose was reduced to an AUC of 4, and olaparib was administered at a dose of 200 mg twice daily for 10 days of a 21-day cycle with a dose intensity of 24%.

In the Velia trial, the most common AEs were thrombocytopenia and nausea occurring in 60 and 80% vs. 30 and 68% of patients in the veliparib-throughout arm and the control arm, respectively. During the combination chemotherapy and maintenance phase, dose reductions and interruptions were higher in the veliparib arm compared to the control group. In particular, the percentage of patients who received veliparib maintenance and discontinued it due to an AE was 19% in the veliparib-throughout group vs. 6% in the control group. The first cause of discontinuation in the veliparib arm was nausea. However, the addition of veliparib to chemotherapy did not affect significantly the patient's quality of life.

The phase III PAOLA-1/ENGOT-ov25 trial explored a combination of a PARP inhibitor in the same clinical scenario (2). Eight hundred six NADOC FIGO stage III–IV patients were randomized to receive the addition of olaparib or placebo to bevacizumab maintenance after complete or partial response to first-line chemotherapy.

Olaparib was used at full dose (300 mg twice daily) for 24 months plus bevacizumab at 15 mg/kg (every 3 weeks) for 15 months, and patients were stratified by *BRCA* status: 237 had tumor *BRCA* mutation and 569 had non-tumor *BRCA* mutation.

The PFS in the ITT population was the primary endpoint, and it resulted to 22.1 months in the olaparib plus bevacizumab group vs. 16.6 months in the placebo plus bevacizumab group, respectively (HR = 0.59; 95% CI 0.49–0.72). Prespecified subgroup analyses demonstrated that *BRCA*-mutated and HRD-positive tumors obtained longer PFS benefit with the combination. In particular, olaparib improved PFS in *BRCA*-mutated patients: 37.2 vs. 21.7 months (HR = 0.31; 95% CI 0.20–0.47), while a small PFS benefit was detected in the *BRCA* wild-type subgroup (18.9 vs. 16.0 months with placebo, HR = 0.71; 95% CI 0.58–0.88). Similarly, HRD-positive patients showed a longer PFS with olaparib (37.2 vs. 17.7 months with placebo, HR = 0.33; 95% CI 0.25–0.45). Median PFS in the HRD-positive without *BRCA* mutation cohort was 28.1 months with olaparib vs. 16.6 months with placebo (HR = 0.43; 95% CI 0.28–0.66), while the HRD-negative subgroup derived no benefit from the addition of olaparib to bevacizumab maintenance (HR = 1.00; 95% CI 0.75–1.35).

Regarding toxicity profile, anemia, and nausea occurred with higher incidence among patients receiving olaparib plus bevacizumab and represented the main reason for treatment discontinuation. However, no clinically meaningful difference in health-related quality of life was observed between treatment arms.

Thus, combinations of chemotherapy or bevacizumab with a PARP inhibitor tend to be more effective in *BRCA* mutant OC patients, similarly to a single-agent PARP inhibitor. However, promising results in the overall population, independently from the HRD status, derive from the combination with the antiangiogenic drug. We speculate that this effect is due to the specific biological effect of bevacizumab in increasing HRD levels in cancer cells (8–10).

## Maintenance Therapy With PARP Inhibitor Single Agent or in Combination With Bevacizumab: Data From PAOLA-1 and SOLO-1 Trials

PAOLA-1 and SOLO-1 trials investigated maintenance therapy with olaparib plus bevacizumab or olaparib alone, after first-line chemotherapy in NADOC patients. The PFS benefit observed with olaparib plus bevacizumab in patients with *BRCA*-mutated tumors (HR = 0.31; 95% CI 0.20–0.47) in the PAOLA-1 study was consistent with the results reported in the SOLO-1 trial (HR = 0.30; 95% CI, 0.23–0.41). However, we note that greater PFS, with regard to the control arm of both trials, was achieved in the PAOLA-1 trial: 21.7 months with placebo plus bevacizumab vs. 13.8 months with placebo in the SOLO-1 trial. This relevant difference could be explained by the effect of bevacizumab in patients with higher disease burden. In fact, PAOLA-1 patients included 35% of cases with macroscopic residual disease after cytoreductive surgery and 30% of stage IV disease vs. 22 and 17%, respectively, in the SOLO-1 trial. Moreover, the SOLO-1 trial did not include patients with HRD-positive tumors without *BRCA* mutations. Thus, the longer PFS obtained by the addition of bevacizumab to olaparib in PAOLA-1 should be interpreted taking into account the lack of a direct comparison with olaparib as single-agent maintenance.

Two previous phase 2 studies showed that the addition of an antiangiogenic agent to a PARP inhibitor prolongs PFS compared to a PARP inhibitor alone in platinum-sensitive relapsed OC patients (10, 11). The NSGO-AVANOVA2/ENGOT-OV24 study compared niraparib and bevacizumab vs. niraparib alone, and the combination arm obtained an improvement in PFS (11.9 vs. 5.5 months, HR = 0.35; 95% CI 0.21–0.57) irrespectively from the HRD status (12). This combination could represent very interesting chemotherapy-free treatment in recurrent setting, if these results will be confirmed in the phase III trial.

## Conclusions

The effectiveness of PARP inhibitors has been evaluated in three different biological subtypes of OC, in first-line setting. *BRCA*-mutated OC patients represent the subgroup obtaining the higher clinical benefit from a PARP inhibitor, and on the basis of phase III trials, chemotherapy plus bevacizumab followed by olaparib plus bevacizumab maintenance would be an advantageous option. Furthermore, the HRD-positive *BRCA* wild type can be considered a new clinical population that showed to derive similar benefit compared to the *BRCA*-mutated subgroup from the addition of a PARP inhibitor and, therefore, the combination of olaparib plus bevacizumab might be an appropriate strategy. Conversely, the HRD-negative population achieved a modest survival advantage from niraparib monotherapy after a response to first-line chemotherapy. Since no comparison with the bevacizumab was performed in the PRIMA trial, for HRD-negative patients, niraparib, or bevacizumab maintenance could be both valid options (13). However, several ongoing studies of new combination strategies (NCT03737643, NCT03602859) could clearly define the role of PARP inhibitors as maintenance therapy in the first-line setting of OC.

As summarized in Table 2, the use of a PARP inhibitor in the first line is effective in the whole population of NADOC, but the magnitude of benefit varies widely among subgroups, highlighting the need to identify specific biological subtypes into clinical practice. In this regard, it is necessary to introduce HRD testing, which is currently expensive and not reproducible in common laboratories. Finally, validated biomarkers to quantify the HRD status in each patient are warranted to identify the subgroup of patients who derived more benefit from PARP inhibitors (14).

**Table 2:** PARP inhibitors in first-line ovarian cancer treatment.

**Trial (enrolled Pts)**	**Key patient population**	**Treatment arms**	**Overall population PFS (months)**	***BRCA*-mutated population PFS (months)**	**HRD-positive population PFS (months)**	**HRD-negative population PFS (months)**
SOLO-1 (391)	*BRCA*-MUTATED NADOC	Olaparib maintenance vs. placebo	–	NR vs. 13.8 HR = 0.30 (95% CI 0.23–0.30) *P* < 0.001	–	–
PRIMA (733)	ALL NADOC	Niraparib maintenance vs. placebo	13.8 vs. 8.2 HR = 0.62 (95% CI 0.50–0.76) *P* < 0.001	22 vs. 10 HR = 0.40 (95% CI 0.27–0.62) *P* < 0.001	21.9 vs. 10.4 HR = 0.43 (95% CI 0.31–0.59) *P* < 0.001	8.1 vs. 5.4 HR = 0.68 (95% CI 0.49–0.94) *P* = 0.020
VELIA (1140)	ALL NADOC	Veliparib + CHT maintenance vs. veliparib + CHT Placebo	23.5 vs. 17.3 HR = 0.68 (95% CI 0.56–0.83) *P* < 0.001	34.7 vs. 22 HR = 0.44 (95% CI 0.28–0.68) *P* < 0.001	31.9 vs. 20.5 HR = 0.57 (95% CI 0.43–0.76) *P* < 0.001	15 vs. 11 HR = 0.81 (95% CI 0.60–1.09)
PAOLA (806)	ALL NADOC	Olaparib + bevacizumab maintenance vs. bevacizumab maintenance + placebo	22.1 vs. 16.6 HR = 0.59 (95% CI 0.49–0.72) *P* < 0.001	37.2 vs. 21.7 HR = 0.31 (95% CI 0.20–0.47) *P* < 0.001	37.2 vs. 17.7 HR = 0.33 (95% CI 0.25–0.45) *P* < 0.001	16.9 vs. 16 HR = 1.00 (95% CI 0.75–1.35)

*NADOC, newly diagnosed advanced ovarian cancer; HR, hazard ratio; PFS, progression free survival; HRD, homologous recombination deficiency; NR, not reached; pts, patients*.

## Author Contributions

EF: conceptualization, methodology, and writing of the original draft. AD: data creation and writing of the original draft. SC: data creation, analysis and interpretation of data, and writing of the original draft. FD, SP, and FC: supervision. MO: conceptualization, data creation, analysis and interpretation of data, supervision, and writing of the original draft.

## Conflict of Interest

The authors declare that the research was conducted in the absence of any commercial or financial relationships that could be construed as a potential conflict of interest.

## References

[1] Moore K Colombo N Scambia G Kim B Oaknin A Friedlander M. Maintenance olaparib in patients with newly diagnosed advanced ovarian cancer. N Engl J Med. (2018) 379:2495–505. 10.1056/NEJMoa1810858 30345884

[2] Ray-Coquard I Pautier P Pignata S Péro D González-Martín A Bergeret R. Olaparib plusbevacizumab as first-line maintenance in ovarian cancer. N Engl J Med. (2019) 381:2416–28. 10.1056/NEJMoa1911361 31851799

[3] González-Martín A Pothuri B Vergote I DePont Christensen R Graybill W Mirza M Niraparib inpatients with newly diagnosed advanced ovarian cancer. N Engl J Med. (2019) 381:2391–402. 10.1056/NEJMoa1910962 31562799

[4] Coleman RL Fleming GF Brady MF Swisher EM Steffensen KD Friedlander M. Veliparib with first-line chemotherapy and as maintenance therapy in ovarian cancer. N Engl J Med. (2019) 381:2403–15. 10.1056/NEJMoa1909707 31562800 PMC6941439

[5] Gray HJ Bell-McGuinn K Fleming GF Cristea M Xiong H Sullivan D. Phase I combination study of the PARP inhibitor veliparib plus carboplatin and gemcitabine in patients with advanced ovarian cancer and other solid malignancies. Gynecol Oncol. (2018) 148:507–14. 10.1016/j.ygyno.2017.12.029 29352572

[6] Lee JM Hays JL Chiou VL Annunziata CM Swisher EM Harrellet MI. Phase I/Ib study of olaparib and carboplatin in women with triple negative breast cancer. Oncotarget. (2017) 8:79175–87. 10.18632/oncotarget.16577 29108297 PMC5668030

[7] Oza AM Cibula D Benzaquen AO Poole C Mathijssen RHL Sonkeet GS. Olaparib combined with chemotherapy for recurrent platinum-sensitive ovarian cancer: a randomised phase 2 trial. Lancet Oncol. (2015) 16:87–97. 10.1016/S1470-2045(14)71135-0 25481791

[8] Franzese E Centonze S Diana A Carlino F Guerrera LP Di Napoli M. PARP inhibitors in ovarian cancer. Cancer Treat Rev. (2019) 73:1–9. 10.1016/j.ctrv.2018.12.002 30543930

[9] Bindra RS Gibson SL Meng A Westermark U Jasin M Pierce AJ. Hypoxia-induced down-regulation of BRCA1 expression by E2Fs. Cancer Res. (2005) 65:11597–604. 10.1158/0008-5472.CAN-05-2119 16357170

[10] Lim JJ Yang K Taylor-Harding B Wiedemeyer WR Buckanovich RJ. VEGFR3 inhibition chemosensitizes ovarian cancer stemlike cells through down-regulation of BRCA1 and BRCA2. Neoplasia. (2014) 16:343–53.e1-2. 10.1016/j.neo.2014.04.003 24862760 PMC4094836

[11] Liu JF Barry WT Birrer M Lee J Buckanovich RJ Fleminget GF Combination cediranib and olaparib versus olaparib alone for women with recurrent platinum-sensitive ovarian cancer: a randomized phase 2 study. Lancet Oncol. (2014) 15:1207–14. 10.1016/S1470-2045(14)70391-2 25218906 PMC4294183

[12] Mirza MR Avall-Lundqvist E Birrer MJ Christensen R Nyvang G Malanderet S Combination of niraparib and bevacizumab versus niraparib alone as treatment of recurrent platinum-sensitive ovarian cancer: a randomized controlled chemotherapy-free study—NSGOAVANOVA2/ENGOT-OV24. J Clin Oncol. (2019) 37(Suppl):5505. 10.1200/JCO.2019.37.15_suppl.5505

[13] Caruso D Papa A Tomao S Vici P Panici PB Tomao F. Niraparib in ovarian cancer: results to date and clinical potential. Ther Adv Med Oncol. (2017) 9:579–88. 10.1177/1758834017718775 29081841 PMC5564880

[14] Banerjee SN Lord CJ. First-line PARP inhibition in ovarian cancer-standard of care for all? Nat Rev Clin Oncol. (2020) 17:136–7. 10.1038/s41571-020-0335-9 32051558

